# Assessing the Performance of CareStart™ Malaria Rapid Diagnostic Tests in Northwest Ethiopia: A Cross-Sectional Study

**DOI:** 10.1155/2021/7919984

**Published:** 2021-10-23

**Authors:** Zelalem Dejazmach, Getaneh Alemu, Mulat Yimer, Banchamlak Tegegne, Abel Getaneh

**Affiliations:** ^1^Department of Medical Laboratory Science, College of Health Sciences, Woldia University, Woldia, Ethiopia; ^2^Department of Medical Laboratory Science, College of Medicine and Health Sciences, Bahir Dar University, Bahir Dar, Ethiopia; ^3^Medical Parasitology, Amhara Public Health Institute, Bahir Dar, Ethiopia; ^4^Medical Parasitology and Vector Control, Dessie Health Science College, Dessie, Ethiopia

## Abstract

**Background:**

While rapid diagnostic tests are an alternative diagnostic tests for microscopy in the diagnosis of malaria in rural settings, their performance has been inconsistent. Performance of rapid diagnostic tests might be affected by manufacturing process, transportation and storage, parasitemia level, and skill of personnel who perform the tests. Therefore, periodic evaluation of the local field performance of rapid diagnostic tests is mandatory in order to make early corrections in case of decreased performance.

**Methods:**

A facility-based cross-sectional study was conducted from January to May 2020 among 257 malaria-suspected patients attending selected health centers in Bahir Dar Zuria district. Capillary blood was collected from each participant and tested for *Plasmodium* infection by CareStart™ rapid diagnostic test kit and thin and thick blood film microscopy. Data were analyzed using statistical software for social sciences version 20 and MedCalc software version 19.3. Sensitivity, specificity, positive and negative predictive values, and kappa value were calculated to evaluate the performance of rapid diagnostic tests against microscopy.

**Results:**

Among 257 study participants, 47 (18.3%) were tested positive for *Plasmodium* infection by at least one of the diagnostic methods. Rapid diagnostic tests revealed 3 false positive and 3 false negative results. The sensitivity and specificity of CareStart Malaria Pf/Pv Combo test were 93.2% and 98.6%, respectively (kappa = 0.918).

**Conclusion:**

CareStart™ rapid diagnostic test has comparable performance with microscopy for malaria diagnosis. We recommend continued use of CareStart Malaria Pf/Pv Combo test at health posts in Ethiopia where microscopy is not available.

## 1. Background

Malaria is a major global health problem which is caused by obligate intracellular protozoan parasites of the genus *Plasmodium* [1]. There are five distinct species that naturally cause malaria in human, namely, *Plasmodium* (*P) falciparum*, *P. vivax*, *P. malariae*, *P. ovale*, and *P. knowlesi* [2]. Malaria caused 228 million cases and 405,000 deaths worldwide in 2018, of which, 93% cases and 94% deaths were from African Region [3]. In Ethiopia, malaria also remains one of the major public health challenges. Approximately 68% of the population is at risk of the disease, and about 1–2 million confirmed cases are reported every year in the country [1, 4]. Among the *Plasmodium* species, *P. falciparum* and *P. vivax* are the two epidemiologically important species in Ethiopia comprising 60% and 40% of infections, respectively. Both species are prevalent in all malaria endemic areas with their relative frequency varying in time and place within a given geographical range [5]. Hence, multispecies rapid diagnostic tests (RDTs) detecting both species have been used in the country.

The World Health Organization (WHO) recommends that antimalarial treatment should be initiated after laboratory confirmation of suspected cases [5–7]. Although microscopic diagnosis of malaria remains the “gold standard,” currently, the use of RDTs has gained major importance wherever microscopy is not available [8]. There are various malaria RDTs that are commercially available. All malaria RDTs detect antigens produced by *Plasmodium* species. Most RDTs are based on the detection of either histidine-rich protein 2 (HRP2), a protein synthesized by *P. falciparum* and lactate dehydrogenase (LDH) or aldolase, enzymes produced by all human *Plasmodium* species [9, 10].

Rapid diagnostic tests have a significant impact on the reduction of malaria burden in the world [11]. They are relatively simple to perform and interpret, and they rapidly provide results, require limited training, and allow for the diagnosis of malaria at the community level [9, 12] without the need of capital investment on electricity but with reasonable sensitivity and specificity [9]. Due to this simplicity, RDTs are being used by health extension workers (HEWs) at health post level in Ethiopia.

Malaria-suspected patients should be confirmed by laboratory diagnosis and treated with efficacious antimalaria drugs within 24 hours of the onset of symptoms. Early diagnosis and immediate treatment is one of the strategies in malaria control and prevention [8]. Diagnosis using gold standard diagnostic technique (blood film microscopy) is indispensable in order to reduce transmission, morbidity, and death due to malaria. This can be done in health institutions equipped with clinical laboratory. In health posts, where there is no laboratory and laboratory professionals, the HEWs are given little training and screen febrile cases with RDT before prescribing antimalaria drugs. While RDTs are an alternative diagnostic tests for microscopy [13], their sensitivity has been inconsistent ranging from 20% to 99% [14]. A few studies have been conducted to assess the performance of RDTs in Ethiopia. The performance of RDTs might vary by lot number, manufacturer, disease transmission intensity, storage, transportation, and end-user performance [15]. Additionally, the accuracy of any RDT depends on concentration of the target antigens in blood [16]. Hence, previous study results do not help to infer for the present situation. Therefore, periodic evaluation of the field performance of RDTs is mandatory to early identify decreased performance and timely corrections. Therefore, the present study is aimed at assessing the performance of malaria RDTs in Bahir Dar Zuria district, where malaria is a public health problem.

## 2. Methods and Materials

### 2.1. Study Design, Area, and Period

A facility-based cross-sectional study was conducted in Yinesa, Andasa, and Robit health centers in Bahir Dar Zuria district, northwest Ethiopia, from January to May 2020. The Bahir Dar Zuria district is one of the 14 districts of West Gojjam Zone, which is located at a distance of 560 km from capital city of the country, Addis Ababa, and the district is situated surrounding Bahir Dar city, capital of Amhara National Regional State. The altitude of the district ranges from 1700 to 2300 meters above sea level [17]. According to Bahir Dar Zuria district health office report for 2019, the district has a total population of 182,730, of whom 93,642 (51.2%) are men and 89,088 (48.8) are women. The district has thirty-six *kebeles*. In the district, there are 9 health centers and 36 health posts. The district receives an average annual rainfall of about 1035 mm. The minimum and maximum temperature lies at 10°C and 32°C, respectively [17]. Likewise, the major transmission season of malaria in the district occurs from September to December after major rainfalls followed by April to May with minor transmission [18].

### 2.2. Sample Size Determination and Sampling Procedure

Sample size for assessment of CareStart RDT performance was determined using the Buderer formula for diagnostic test studies [19].

(1) $$\begin{array}{c} \text{Study participants}:n=\frac{\left(Z^{2}\text{SN}\left(1-\text{SN}\right)\right)}{d^{2}P}, \end{array}$$

where *n* = *sample* *size*, *z* = 95%*statistic* for *level* of *confidence* (*Z* = 1.96), *SN* (*sensitivity*) = 95.4% from previous study from Sheraro, northwest Ethiopia [20], *P* (previous malaria prevalence) = 13.4% from previous study at Sheraro, northwest Ethiopia [20], *d* = *margin* of *error* *tolerated* (*d* = 0.07), and *n* = ((1.96)^2^ × 0.954(1 − 0.954))/(0.07)^2^ (0.134) = 257.

Therefore, a total of 257 malaria-suspected patients were included in the study to evaluate the test performance of CareStart™ RDTs against microscopy. Three health facilities, Andasa health center, Yinesa health center, and Robit health center, were selected using simple random sampling technique among nine health centers. On average, 214, 198, and 142 febrile patients visited the laboratory at Yinesa, Andasa, and Robit health center laboratories, respectively, per two months from January to March in 2019. Therefore, by proportional allocation, 100, 92, and 65 patients were allocated in Yinesa, Andasa, and Robit health centers, respectively. Then, systematic random sampling was used to select study participants, and the sampling interval (*k* value) was 2. Of the first two participants, one patient was randomly selected by lottery method, and then, every 2^nd^ patient was selected to participate in the study.

The study participants were all self-presenting clinically suspected malaria patients during data collection period, whose age were greater than or equal to 1 year old, who were attending at three randomly selected health facilities. Children below the age of one year, critically ill patients who were unable to respond to research questions, individuals who had taken antimalaria drugs within 4 weeks prior to data collection, and individuals who had taken antibiotic or any antipain drugs within 24 hours prior to data collection were excluded.

### 2.3. Data Collection

Sociodemographic and related data were collected through face to face interview before blood sample collection by trained laboratory technicians using semistructured questionnaires prepared in English and translated to Amharic. Each febrile patient was sampled with capillary blood and used for *Plasmodium* examination using RDT, thick and thin smear microscopy. Blood films were prepared, dried, stained, and examined for *Plasmodium* parasitemia following standard protocol [9, 12]. RDTs were also run following the manufacturer's instructions. 5 *μ*l of blood sample was added onto the test device window using the specimen transfer device (micropipette) provided with the kit, followed by adding 2 drops (60 *μ*l) of reagent buffer on the buffer well. Results were read 20 mins after addition of the sample and buffer [21].

### 2.4. Statistical Analysis

Data were checked for completeness and entered and analyzed in Statistical Package for Social Science software version 20. Descriptive statistics was manipulated to explain the study participants and to show the malaria prevalence by CareStart™ RDT and microscopy. Performance of RDTs was measured by calculating sensitivity, specificity, positive predictive value (PPV), and negative predictive value (NPV) against microscopy result using MedCalc software version 19.3. Kappa value was also calculated to show the strength of test agreement between “RDTs and microscopy.” Kappa value was interpreted as follows: values = 0 as indicating no agreement, 0.01–0.20 as none to slight, 0.21–0.40 as fair, 0.41–0.60 as moderate, 0.61–0.80 as substantial, and 0.81–1.00 as almost perfect agreement [22].

### 2.5. Ethical Considerations

The research was carried out after ethical approval was obtained from the Institutional Review Committee of College of Medicine and Health Sciences, Bahir Dar University. Additionally, supportive letters were obtained from Amhara Public Health Institute, West Gojjam Zone health department, and Bahir Dar Zuria district health office, and permission was obtained from each health center authority. Information obtained at any course of the study was kept confidential. Informed verbal consent was obtained from each participant and assent from guardians for children under 18 years old. Moreover, no personal and health facility identifier was included as participants were given a unique code. Malaria-suspected patients who were positive for malaria or other haemoparasites by any of the tests were linked to the respective health center for appropriate treatment.

## 3. Results

### 3.1. Sociodemographic Characteristics of Study Participants

Among a total of 257 febrile patients participated in the present study, 151 (58.8%) and 106 (41.2%) were males and females, respectively. The participants' age ranged from 1 to 82 years with mean age of 21.0 (±17.1 SD) years. All the study participants were rural inhabitants. Regarding to their educational status, 194 (75.5%) were unable to read and write and the rest 63 (24.5%) were able to read and write or have attended formal education (Table 1).

### 3.2. Malaria Prevalence and Parasitemia Level

Among 257 participants, 47 (18.3%) were tested positive for *Plasmodium* infection by at least one of the diagnostic methods (RDT or microscopy). Each test separately detected *Plasmodium* infections in 44 (17.1%) participants. Blood film microscopy results revealed that 30 (11.7%), 11 (4.3%), and 3 (1.2%) participants were positive for *P. vivax*, *P. falciparum*, and mixed infections, respectively. CareStart^TM^ RDT detected *P. vivax*, *P. falciparum*, and mixed infections in 32 (12.5%), 10 (3.9%), and 2 (0.8%) participants, respectively (Table 2). The mean asexual stage parasite density of microscopy-confirmed cases was 4557.29 (±4032.5 SD) with a range of 80 to 16480 parasites/*μ*l, and 30 (68.2%) of the infections had parasite density greater than 2000 parasites/*μ*l. There were two cases that had a parasitic load less than 200 parasites/*μ*l, which were negative by RDTs.

### 3.3. Comparison of CareStart™ against Microscopy

Among 44 positive results by microscopy, CareStart™ malaria RDT detected 41 samples as positive. Three samples were false negative by RDT as 1 and 2 of them were positive for *P. falciparum* and *P. vivax*, respectively, by microscopy. On the reverse, 3 samples were false positive by RDT as microscopy results were negative (Table 3).

### 3.4. Diagnostic Performance of CareStart™ RDT versus Microscopy

The sensitivity, specificity, PPV, and NPV of RDTs in detecting overall *Plasmodium* infection were 93.2%, 98.6%, 93.2%, and 98.6%, respectively. Almost perfect measure of test agreement was found between RDT and microscopy with kappa value of 0.918. However, the test agreement was 0.394 in detecting mixed infection (Table 4).

## 4. Discussion

Comparison of RDTs' performance against blood film microscopy in the present study revealed sensitivity, specificity, PPV, and NPV of 93.2%, 98.6%, 93.2%, and 98.6%, respectively. This result showed that the sensitivity of RDT in detecting *Plasmodium* infection was in line with findings from Kola Diba, northwest Ethiopia (95.0%) [23], Mayani hospital, northwest Ethiopia (95.4%) [20], Serbo, southwestern Ethiopia (95.8%) [24], Kenya (93.4%) [25], and China-Myanmar (89.7%) [26]. On the other hand, it was lower than reports from Wondogenet, northwest Ethiopia (99.4%) [27] and Felegeselam, northwest Ethiopia (99.8%) [28]. Such variations could be due to quality of different RDT products which may differ in detection limits, sometimes even within the same country [29], lot-to-lot variation [30], parasite load [31], observer variation, lower transmission intensities [32], mutation, or deletion in the HRP 2/3 genes [33]. The RDT performance would be higher if data was collected during the major transmission season.

The attempt to evaluate the performance of the CareStart Malaria Pf/Pv Combo test to diagnose *P. falciparum* infections indicated the low level of sensitivity (81.8%) which was comparable with previous report in Jimma, southwest Ethiopia (85.6%) [34]. However, this figure is lower than previous studies reported from Felegeselam Health Center, northwest Ethiopia (99.7%) [28], Mayani hospital, northwest Ethiopia (94.4%) [20], in Kola Diba, northwest Ethiopia (92.9%) [23], Serbo, southwest Ethiopia (96.4%) [24], and China-Myanmar (88.52%) [26]. The sensitivity of CareStart™ RDT for the detection of *P. vivax* (93.3%) was in consistent with the study conducted in Kola Diba, northwest Ethiopia (90.9%) [23], Serbo, southwest Ethiopia (95.3%) [24], and China-Myanmar (88.5%) [26]. Likewise, it was lower than results of a study from Felegeselam, northwest Ethiopia (99.7%) [28]. On the other hand, it was higher than findings from Jimma, southwest Ethiopia (85.0%) [34]. This difference might be due to the above aforementioned reasons for *Plasmodium* infections [30, 31, 33]. The sensitivity (33.3%), PPV (50.0%), and kappa value (0.394) of RDTs in detecting mixed infections in the present study is low. This might be due to low parasitemia level that two RDT negative samples had a parasitemia level < 200/*μ*l while all positive samples by both RDT and microscopy had a parasitemia level of >4000/*μ*l. Moreover, identification of mixed infection by microscopy is also prone to reader errors and needs microscopy experts. Hence, large-scale studies recruiting reasonable number of patient samples with mixed infection and including more sensitive molecular methods are recommended to give definitive conclusion on the performance of RDTs in detecting mixed infections.

Based on the WHO limit of detection (specificity > 90%), specificity of 98.6%, 99.6%, and 98.2% for overall *Plasmodium*, *P. falciparum*, and *P. vivax*, respectively, revealed that RDTs could be used for malaria diagnosis at health post and other public health facility levels in the absence of microscopy [35]. The specificity of the current study for *P. falciparum* and *P. vivax* (99.6% and 98.2%, respectively) was comparable with previous findings at Felegeselam in northwest Ethiopia (97.8% and 99.9%) [28], Serbo, southwest Ethiopia (100% and 100%) [24], and China-Myanmar (98.26% and 100%) [26]. The specificity of the present study for diagnosis of *Plasmodium* species was comparable with study done at Felegeselam in northwest Ethiopia (97.7%) [28], Kola Diba, northwest Ethiopia (94.2%) [23], Mayani hospital, northwest Ethiopia (99.3%) [20], and China-Myanmar (98.3%) [26]. Likewise, the finding of this study was found higher than study done in Butajira, southwest Ethiopia (82.7%) [16] and western Kenya (76.9%) [25]. Such variations in specificity within the same test could be explained by variation in species distribution across study sites, the persistence of *P. falciparum*-specific HRP2 in patients who had been treated [9], and the sequestration of the parasites in the deep organs to reduce circulating parasites [36].

The RDTs had high NPV (98.6%) and PPV (93.2%) in the present study, which was relatively consistent with a study conducted at Felegeselam Health Center in northwest Ethiopia (99.7% and 97.8%) [28], Kola Diba Health Center, northwest Ethiopia (96.7% and 91.3%) [23], and Mayani hospital, northwest Ethiopia (98.9% and 99.6%) [20], respectively. This indicates the ability of RDTs to correctly diagnose malaria negative and positive patients was high, meaning that it was reliable in ruling out malaria, and also, infected patients will be correctly diagnosed as positive for malaria and avoid unnecessary treatment.

## 5. Conclusions

CareStart™ RDT had very good sensitivity and specificity for malaria diagnosis with an excellent agreement with the reference blood film microscopy. We recommend continued use of CareStart Malaria Pf/Pv Combo test at health posts in Ethiopia where microscopy is not available. However, the test performance in detecting light infections should be evaluated against a more sensitive molecular technique.

### 5.1. What Is Already Known on This Topic

- Different factors affect performance of rapid diagnostic tests

### 5.2. What This Study Adds

- Previous study results do not help to infer for the present situation

## Figures and Tables

**Table 1:** Sociodemographic characteristics of malaria-suspected cases in Bahir Dar Zuria district, northwest Ethiopia, from January to May 2020.

Characteristics	Categories	Frequency	Proportion (%)
Sex	Male	151	58.8
	Female	106	41.2

Age group (in years)	1-5	75	29.2
	6-15	37	14.4
	>15	145	56.4

Educational level	Unable to read and write	194	75.5
	Able to read and write	12	4.7
	Primary	33	12.8
	Secondary and above	18	7.0

Primary occupation	Farmer	224	87.2
	Employed	3	1.2
	Student	22	8.6
	Housewife	8	3.1

Marital status	Single	128	49.8
	Married	129	50.2

Family size	<5	128	49.8
	≥5	129	50.2

**Table 2:** Prevalence of malaria by CareStart™ RDT and blood film microscopy in Bahir Dar Zuria district, northwest Ethiopia, from January to May 2020.

Result	Diagnostic methods		Combined result
	Care Start™	Microscopy	
*P. falciparum*	10 (3.9%)	11 (4.3%)	12 (4.7%)
*P. vivax*	32 (12.5%)	30 (11.7%)	32 (12.5%)
Mixed infection	2 (0.8%)	3 (1.2%)	3 (1.2%)
Total positive	44 (17.1%)	44 (17.1%)	47 (18.3%)
Negative	213 (82.9%)	213 (82.9%)	210 (81.7%)

**Table 3:** Comparison of CareStart™ RDT results with microscopy among malaria-suspected cases attending selected health centers in Bahir Dar Zuria district, northwest Ethiopia, from January to May 2020.

Test		Microscopy				
		*P. falciparum*	*P. vivax*	Mixed infection	Negative	Total
CareStart*™* RDT	*P. falciparum*	9	-	-	1	10 (3.9%)
	*P. vivax*	1	28	2	1	32 (12.5%)
	Mixed infection	-	-	1	1	2 (0.8%)
	Negative	1	2	-	210	213 (82.9%)
	Total	11 (4.3%)	30 (11.7%)	3 (1.2%)	213 (82.9%)	257 (100%)

**Table 4:** Diagnostic performance of CareStart™ RDT against microscopy in Bahir Dar Zuria district, northwest Ethiopia, from January to May 2020.

Parasites	Sensitivity (%)	Specificity (%)	PPV (%)	NPV (%)	Kappa value
*P. falciparum* monoinfection	81.8	99.6	90.0	99.2	0.851
*P. vivax* monoinfection	93.3	98.2	87.5	99.1	0.890
Mixed infection	33.3	99.6	50.0	99.2	0.394
Overall *Plasmodium* infection	93.2	98.6	93.2	98.6	0.918

## Data Availability

The questionnaire and SPSS data used to support the findings of this study can be found from the corresponding author.

## Abbreviations

HEWs: Health extension workers
HRP2: Histidine-rich protein 2
LDH: Lactate dehydrogenase
NPV: Negative predictive value
PPV: Positive predictive value
RDT: Rapid diagnostic test
WHO: World Health Organization.
